# The impact of travel live streaming on tourists’ travel intention on the spot

**DOI:** 10.1371/journal.pone.0333811

**Published:** 2025-10-09

**Authors:** Zheng Zhang, Mengjie Qin, Zihui Li, Hao Zhu

**Affiliations:** School of Management, Wuhan Textile University, Wuhan, Hubei, China; MCC Boyd Tandon School of Business, INDIA

## Abstract

The objective of this study is to examine the mechanisms that influence tourists’ intentions to travel in the context of live streaming. The utilization of text mining techniques in this research endeavor has yielded two critical dimensions, namely, the characteristics of live streamers and the attributes of live streaming content, as discerned from user comments. The present study proposes a model grounded in the Stimulus-Organism-Response (SOR) theoretical framework. Subsequently, a series of questionnaires were disseminated through various digital platforms, including WeChat, QQ, and TikTok. This endeavor yielded a total of 511 valid responses from audiences within China’s travel live streaming sector. Subsequently, structural equation modeling (SEM) was employed to analyze the data. The findings indicate that live streamer characteristics (e.g., professionalism, image, charm), live streaming content characteristics (e.g., tourist destination information), perceived emotional value, and perceived utilitarian value can directly influence tourists’ intention to travel on the spot. Furthermore, live streamer characteristics and live streaming content characteristics directly impact perceived emotional value and perceived utilitarian value, and indirectly influence travel intention through double intermediaries.

## 1. Introduction

Tourism is a prevalent form of leisure for many individuals, offering a means of relaxation. The consumption of spiritual products has long been a driving force behind the tourism sector. According to the report of “Travel Live Times”. Since April of 2020, there has been a 100% increase in the number of users watching travel live streaming, as well as an increase in the duration of these viewings. AI Media Consulting’s research report on online live streaming revealed that in 2020, the number of users in China who watched live streaming reached nearly 524 million. Among these, travel live streaming, an unconventional form of live streaming, exhibited considerable development potential. It was also called “the first year of travel live streaming” by many people in the industry in 2020 [1]. Since 2021, various entities—including online travel agencies (OTAs), destination official agencies, travel agencies, platform enterprises (e.g., TikTok, Kwai, Taobao), online celebrity talents, tour guides, and other institutions. Individuals—have collaboratively promoted the integration of “live streaming” and “tourism”. This initiative has been undertaken to capitalize on the vast potential of China’s live streaming market, thereby enhancing the live streaming capabilities of content creators. The strategic combination of these elements has fostered the collaborative development of both online and offline field travel for tourists. In light of the pervasive influence of live streaming in contemporary society and the subsequent surge in tourism following the epidemic, numerous scholars and industry practitioners have expressed a keen interest in exploring the intricate relationship between live streaming and tourist travel intention within the tourism industry, encompassing a comprehensive array of dimensions and facets. However, in recent two years, research on travel live streaming has been limited, with few related studies addressing the complex factors influencing live streaming, tourists, and travel intentions. Previous studies have focused on purchase intention [2]and participation intention [3]. Therefore, a comprehensive examination of the influence mechanism of travel live streaming on travel intention is imperative.

Travel live streamer are an indispensable part of live streaming [4], live streamer characteristics have been studied in the field of live streaming, such as live streamer language style, credibility, attractiveness, professional knowledge [5,6]. Research has demonstrated that the characteristics of live streamers can influence consumer behavior choices. It is evident that live streamers are capable of meeting consumers’ needs by providing a comprehensive explanation and visual representation of products. Concurrently, they can furnish practical suggestions and guidance to consumers, empowering them to make more informed decisions. Consequently, it is imperative to examine the impact of live streamer characteristics on consumers’ preferences. However, there is a paucity of studies that have examined the characteristics of live streamers in the tourism sector, particularly from the perspective of tourists, using text mining and questionnaires. This paper explores three dimensions of live streamer characteristics: professionalism, image charm, and popularity. Consequently, this constitutes the primary research content of this paper.

The advent of live streaming has profoundly transformed the landscape of communication, facilitating unprecedented interactions among media entities and their audiences. This technological advancement has not only augmented audience participation but also fostered a sense of collective existence, thereby enhancing trust among members [7,8]. In the context of travel live streaming, this technology has been shown to facilitate improved situational awareness among tourists, providing insights into the latest developments at their destination, notable attractions, and unique experiences. This, in turn, has been demonstrated to enhance the immersive experience of tourists, potentially influencing their travel intentions [9]. However, there is a paucity of research on the integration of live streaming content characteristics and tourist travel intention on the spot. This study utilizes a multifaceted approach encompassing text mining, complemented by a comprehensive exploration of tourist travel intentions in real-time settings. It delves into two primary research dimensions: the impact of live streaming on users’ perceptions and the characteristics of live streaming content pertaining to tourist destinations. These dimensions constitute the second research focus of this paper.

In addition, in marketing, the perceived value used to judge consumers’ buying behavior is essential for users [10]. The following text is intended to provide a comprehensive overview of the subject matter. As indicated by the findings of preceding studies, when individuals confronted with a multitude of service or product alternatives, perceived value emerged as a pivotal element in their selection process. When the perceived value is higher, users are more interested in buying a product [11]. Different scholars have discussed the dimensions of perceived value from many angles, including functionality, emotion and social value [12,13]. Furthermore, scholars have indicated that perceived value can function as an intermediary variable, thereby establishing a connection between live streaming, tourists, and intention [14,15]. Despite the extensive research on perceived value as an intermediary variable, there is a paucity of studies on establishing a SOR model of live streaming, live streamer, and travel intention with two intermediaries of perceived emotional value and perceived utilitarian value in the field of tourism. The model under consideration takes live streamer characteristics and live streaming content characteristics as S, double intermediaries as O, and travel intention as R, which is the third research content of this paper.

The research value of this study is as follows: Theoretically, the characteristics of live streamers and live streaming content that affect tourists’ intentions are mined from the perspective of tourists through text mining, in combination with previous literature. The second step involves the division of perceived value into two dimensions: perceived emotional value and perceived utilitarian value. This division is followed by an exploration of the mediating role of this factor in the research model. Thirdly, in accordance with the SOR model, the three dimensions of live streamer characteristics and the two dimensions of live streaming content characteristics must be established and verified. Additionally, the research model of perceived value on tourist travel intention on the spot must be developed. The practical value of this paper lies in its provision of enhanced recommendations for the managers and operators of the tourism industry.

## 2 Literature review

### 2.1 Travel live streaming

Travel live streaming generally combines “tourism” and “live streaming”. Live streaming is a new social media format that integrates communication technologies to transmit information in real time through audio, video, and text [16,17]. Travel live streaming involves recording travel experiences in real time and broadcasting them on streaming media channels or social media platforms. This allows tourists to interact with travelers and generate the idea of field travel during the interaction process [16]. Compared with traditional tourism promotion methods, travel live streaming eliminates the need for offline marketing, overcomes the limitations of using words and pictures for online marketing, and meets consumer needs due to its powerful interactivity [18]. In addition, travel live streaming can also let people enjoy the beautiful scenery at home, understand the relevant content, and enhance their happiness [19], Share the entertainment that tourists need most with live stream viewers [20], In order to stimulate tourists to produce or enhance travel intentions on the spot.

A live streamer plays an important role as a spokesperson [4]. Similar research on live streamers exists in many fields, including celebrity, KOL, and brand live streamers [4]. Recent tourism research shows that live streamers’ characteristics can affect tourists’ travel intentions. For instance, a live streamer can persuade their audience and increase their desire to travel by using the right language [6]. In digital tourism, attraction and credibility will affect the audience’s perception of live streamer, but the presentation of live streamer’s professional knowledge will significantly affect the trust of live streamer, and affect the audience’s loyalty and intention to cooperate with live streamer [5]. The research also shows that in live streaming tourism, professional tour explanation, interactive experience and in-depth presentation of immersive experience can attract customers better and effectively promote the sustainable development of live streaming tourism [5].

Currently, many studies examine the marketing methods of live streaming. Some of these studies explore the impact of live streaming content on consumers [21]. For example, good live visual effects can improve the fun of tourists [22], thus promoting tourists’ intention to participate [23]. At the same time, some scholars put forward the viewpoint of mixed tourism experience, which can be used to supplement the shortcomings of travel live streaming at this stage [24]. The other part of the research is devoted to studying the influence of virtual reality on consumers’ intention to visit from the aspects of presence and visual information. Research shows that virtual experience can enhance the sense of reality of tourists in virtual scenes and improve their satisfaction and loyalty [25]. Some telepresence, such as the sense of reality, immersion and existence in the virtual environment, has a positive impact on tourists’ intention to visit the destination on the spot [26], tourism organizations can also directly and effectively influence users’ opinions and decisions through this interaction, experience and immersion [27]. At the same time, visitors can also experience the destination in advance with the help of virtual reality, preview the local attractions, and plan the travel planning process and activities in advance [28].

### 2.2 Travel intention

Scholars’ research on intention mainly focuses on consumers’ intention to buy in the field of e-commerce. In the online environment, purchasing intention is used to measure the degree to which customers are willing to buy goods or services through online platforms [29]. Craig [30] was the first to introduce the concept of intention into the field of tourism, believing that intention is a decision to make a certain behavior through subjective feelings and perceptions of future states. With the deepening of research, the concept of intention has also developed in the field of tourism. Chen & Tsai [31] defined tourism intention as the behavior of tourists who will revisit their destination and are likely to recommend attractions to others. In the study of travel intention, it is found that there are many factors affecting travel intention at this stage, and the external factors are: reputation of scenic spots, destination image, tourists’ attitude, city image, etc. [32,33], while internal factors mainly involve the influence of tourists’ subjective perception and psychological state on their travel intention, such as flow experience and trust [34], psychological image [26], flow experience and destination attachment [35].

### 2.3 Perceived value

Due to the background of different disciplines, perceived value is endowed with different connotations. Richins applied perceived value to marketing, including both emotional and rational aspects, to judge consumer purchasing behavior [10]. Previous studies have found that when users are faced with a variety of services or product choices, perceived value becomes one of their choice references. Zeithaml [36] based on customer psychology, pointed out that perceived value is generated by comparing cost and evaluating utility, and links price, perceived quality and perceived value, and proposed a model of price-perceived quality-perceived value based on this. Currás-Pérez et al.[37] pointed out that perceived value is a comprehensive subjective evaluation by users to determine whether a product meets their needs, but these cannot fully capture the perceived value of tourists. Later, scholars divided perceived value into different measurement dimensions, including utilitarian, emotional, holistic, cost-effective, social, novelty, economic, social and other dimensions [12,38–40]. The study found that in the field of tourism, if the tourist destination provides a higher perceived value to tourists, tourists will tend to travel on the spot, which will also make the travel experience more enjoyable. When exploring the role of perceived value, it is also found that perceived value can connect the relationship between before and after variables and play an intermediary role, which has been verified by the intermediary effect. (for example, Zhai & Chen [15] and Lim et al. [14]).

### 2.4 SOR theory

From the perspective of environmental psychology, Mehrabian & Russell [41] first proposed a SOR theory named “stimulus-organism-Response”, which included the factors of internal cognition and emotional activities, that is, the “organism” factor, and deeply studied people’s internal response. Many studies have focused on stimulus factors, and found that external stimulus sources can exist in many different forms, not only in a single form, but also in environmental factors and interpersonal related factors [42]. Previously, SOR model has been widely used in online consumer behavior research [43,44]. At present, SOR theory is also widely used in the study of tourist behavior in tourism literature [45–47], there are also studies that apply SOR theory to the influence of travel intention [48]. Generally speaking, the concept and application of SOR theory have been verified and widely used in various studies. In this study, SOR theory is used to study the influence of two dimensions on consumers’ intention to travel in travel live streaming, which provides a theoretical basis for explaining how external environmental stimuli affect consumers’ perceived value and thus their intention to travel.

### 2.5 Research design and model construction

#### 2.5.1 Travel live streaming characteristics identification.

To obtain more accurate information about popular topics and tourist demands, this paper uses Octopus software to collect relevant data from comments made by tourists during live streams of trips to Shanxi and Gansu selected by East to See the World. A total of 33,578 valid comments were collected in this article. Due to the large amount of unstructured text data, this paper uses Python software to preprocess and segment the comments. The Jieba tool is then used to generate word frequency statistics and deeply mine the text information of online comments. Use Jieba tools to perform stop word filtering, preprocessing, and word segmentation for text comments. For example, duplicate characters, user IDs, special characters, etc., and delete useless text comments in the data, such as “you”, “what”, “he”, etc., and uniformly process short texts with the same meaning in the comment text.

To further reveal the relationships and patterns in the comments, Python was used to construct a social network of comments on travel livestreaming. Then, Gephi was used to perform a social network diagram and cluster analysis to identify key themes and viewpoints in the comments, as well as their interrelationships. Seven modules were obtained in the end. According to Gephi’s modular analysis, keywords with similar meanings in modules 0, 1, and 5 that were mentioned many times were summarized as characteristics of live streamers in travel live streaming. Keywords with similar meanings that appeared frequently in modules 2, 3, 4, and 6 were summarized as content characteristics in travel live streaming.

Based on observations of original tourist comment data and analysis of the social network diagram, this paper classifies 300 keywords according to semantics and part of speech.Based on the observation of the original data of tourists’ comments and the analysis of social network diagram, this paper classifies 300 keywords according to their semantics and parts of speech.

According to the classification of live streamer characteristics and keywords obtained from Table 1, the keywords can be divided into three dimensions based on the social network diagram’s classification of keywords and parts of speech: “Professionalism”, “image charm”, and “popularity”. Table 2, hows that the characteristics of live streaming content are divided into two dimensions: “Live Streaming Rendering Effect” and “Tourist Destination Information”. Table 3, shows that perceived value is divided into “perceived utilitarian value” and “perceived emotional value”. Finally, an analysis of the relationships between words reveals that professionalism, image charm, popularity, live streaming rendering effect, tourist destination information, perceived utilitarian value, perceived emotional value, and intention to travel on the spot are related.

**Table 1:** Keyword classification of live streamer characteristics.

category	keyword		
	noun	adjective	verb
professionalism	Ability, language, content, eloquence, speech, status, strength, atmosphere, live room, platform, dialogue, free, discount, price, ticket, history, culture, cultural tourism, story, goal, method, map, itinerary, destination, route	Vivid, professional, dedicated, really good, excellent, awesome, awesome, enthusiastic, expensive, cheap	Explain, accompany, scatter flowers, chat, respond, experience, plan
Image charm	Soul, character, character, image, ordinary people, young people, young men, charm, clothes, courage, reserves, vision, literary style, cultural heritage, heritage, poetry and books, language, knowledge, mind, ideas, common sense, thoughts, thinking, emotional intelligence, pattern	Cute, singing well, good-sounding, imposing, funny, atmospheric, polite, good-looking, handsome, witty, erudite, talented, sparkling, connotation, height	Fear of heights, singing, reading, exporting chapters, reading thousands of books, and traveling thousands of miles
Popularity	Dong Yuhui, Shi Ming, mayor, expert, live streamer, commentator, Dean Zhang, Guan Chang, curator, fans, Teacher Shi, mother-in-law	Achievements, role models	

**Table 2:** Keyword classification of live streaming content characteristics.

category	keyword		
	noun	adjective	verb
Live streaming rendering effects	Picture, Lens, Photographer, Environment, Screen, Sound, Playback, Photography, Colorful, Sky	authentic	montage
Tourist destination information	Shanxi, Gansu, Wutai Mountain, Yan men Pass, Taiyuan, Wu Wei, Jia Yu guan, Datong, Ancient City, Hanging Temple, Hexi Corridor, Zhang ye, Hengshan, Da yuan, Ding xi, Hun yuan, Jin Ancestral Hall, Grottoes, Baita Mountain, Grassland, Ying xian, Desert, Yuncheng, Tian shui, Museum, Ying xian Wooden Tower, Jiu Quan, Hong dong Big Locust Tree, City Wall, Yun gang Grottoes, Five Lakes and Four Seas, Dai County, Ruins, Xiang fu, Imperial City, Chaka Salt Lake, Xin Zhou, Products, Jade, Wang’s, Wang’s, Food, Beef Noodles, Specialties, Agricultural Products, Liang fen, Coffee, Chicken Thighs, Lanzhou Ramen, Ramen, Beef, Gu Cheng Yogurt, Lanzhou Beef Noodles, Pork Belly, Taste, Links, Cultural Relics, Camels, Programs, Scenes, Silk Scarves, Dances, Rituals, Craftsmen, Generals, Princess Wencheng, Physical Strength, Strength, Body	Authentic, distinctive, traditional	Inheritance, selling, appreciating, receiving blessings, climbing, praying, representing, visiting, relative, experiencing, Camel riding, horseback riding

**Table 3:** Classification of perceived utilitarian value and perceived emotional value keywords.

category	keyword		
	noun	adjective	verb
Perceived utilitarian value	Time-saving, value, importance, economy, education, scenery, beauty, scenic spots, geography, place, homeland, Buddhism, architecture, information, humanities, peace, China, motherland, country, nation, faith, life, soul, sublimation, spirit, dream, human heart	Useful, satisfying, critical, wonderful, convenient	Travel, live streaming, watching, influence, gratitude
Perceived emotional value	Happy, feelings, feelings, moods, truths, values, interests, attitudes, hearts	Hahaha, laughing to death, warm, interested, attentive, proud	Tears, thoughts, tears, admiration, empathy, belief, experience

#### 2.5.2 Model construction.

According to the literature review and data mining analysis, this study proposes a research model based on the SOR model. This model considers professionalism, image charm, popularity, the live-streaming rendering effect, and tourist destination information as stimulating factors that affect perceived utilitarian and emotional values. Ultimately, these factors influence tourists’ travel intentions. The model emphasizes the relationships between various structures.

Professionalism means that live streamer has relevant professional knowledge and skills and can accurately introduce products [49]. Bansal & Voyer [49] believed that consumers trust the opinions of professionals more, so the more professional live streamer, the more it can influence consumers’ decision-making. The results show that, compared with attractiveness and credibility, the professional knowledge of tourism live streamer has the strongest influence on consumers, and has a great influence on trust [5], and participating in and perceiving the brand value of the destination have a positive impact [50]. When live streamer has relevant knowledge and skills, it can bring more learning, guidance and suggestions to the audience [5]. At the same time, live streamer attracts the audience’s attention by responding to the audience’s requests quickly and providing travel-related knowledge, and strengthens the audience’s responsiveness and travel intention [35]. Professional travel live streamer has rich travel experience and corresponding knowledge reserves, which can help tourists plan their trips better and provide them with practical travel strategies, scenic spots recommendations, etc., which will help improve tourists’ satisfaction and enhance their intention to travel.

**Hypothesis 1.** The professionalism of travel live streamer has a positive impact on tourists’ **(a)** perceived utilitarian value.**(b)** perceived emotional value.**(c)** travel intention on the spot.

The image charm of a travel live streamer can attract tourists [51], travel live streamer attracts the audience through a series of qualities such as excellent appearance, humorous language, unique behavior, etc. This attraction can usually make the audience invest more feelings, establish a deeper perceptual connection, and further improve the emotional expectation of the travel experience [5]. The external image and internal quality of live streamer can also stimulate the emotional attachment of users, and then have a positive impact on the gift-giving intention [52]. Through its unique personal charm, expressive skills and interactive ways, travel live streamer can establish emotional connection with tourists and help them make travel plans. Based on the relevant research of scholars, it can be predicted that the image charm in this study has a positive impact on perceived value and travel intention.

**Hypothesis 2.** The image charm of travel live streamer has a positive impact on tourists’**(a)** popularity utilitarian value.**(b)** perceived emotional value.**(c)** travel intention on the spot.

McCracken meaning transfer model explains the process by which online characters influence how consumers make choices [53]. The model holds that the behavior characteristics of travel live streamer can give them a better image, such as their social status and reputation. Fame refers to live streamer’s personal influence and his position in the industry [54]. According to the research, effective celebrity endorsements can improve the popularity of destinations, improve personal perception of destinations, and then promote tourism products and services and enhance consumers’ intention to travel [55,56]. The more well-known live streamer enhances the audience’s intention to travel through information, entertainment and interaction, and this influence is further amplified in travel live streaming [4]. When the tourism live streamer and tourism information appear in the webcast room at the same time, the tourism live streamer and tourism products will be combined with each other, which will make tourists feel their own pleasure and further generate the idea of field travel.

**Hypothesis 3.** The popularity of travel live streamer has a positive impact on tourists’**(a)** perceived utilitarian value.**(b)** perceived emotional value.**(c)** travel intention on the spot.

By providing real and rich information and interactive experience, travel live streaming can help tourists better understand their destinations, reduce the difficulty of decision-making in actual travel and reduce search time [52]. For example, live streaming shows the actual situation of scenic spots, special services and local cultural characteristics, and a large number of detailed and rich videos and pictures can help tourists better identify the quality of recommended products [2]. A good live tour with goods will produce good results, especially in terms of vision and pictures and visual appeal, which can make individuals feel happy [22,57], which has a continuous positive viewing impact on the users watching the live tour [58], thus promoting the intention of tourism consumers to participate [23]. Therefore, the presentation effect of live streaming content has a positive impact on perceived utilitarian value, perceived emotional value and travel intention.

**Hypothesis 4.** The rendering effect of travel live streaming content has a positive impact on tourists’ **(a)** perceived utilitarian value.**(b)** perceived emotional value.**(c)** travel intention on the spot.

Tourist destination resources include natural resources, human resources, scenic spot structure, architectural style and artworks of tourist destinations. The research shows that the immediacy of the destination during travel live streaming has a positive impact on tourist travel intention on the spot by affecting consumers’ trust [48]. Through live streaming platform, tourists can know the latest situation of destinations, scenic spots or special experiences at the first time, and this transparency is closely related to the increase of consumers’ wishes [9]. Research shows that live streaming function of destination can increase consumers’ travel intention through their trust and presence, so we should pay attention to improving the fluency, clarity and interactive response time of live video to ensure the authenticity of the content displayed in live streaming [48]. Travel live streamer convey real information to the audience, thus attracting them, stimulating their interest and encouraging them to visit these destinations. Based on the study of tourism-related literature, it can be predicted that the information of tourist destinations in this study has the following relationship with perceived value and travel intention.

**Hypothesis 5.** Tourism destination information of travel live streaming content has a positive impact on tourists’ **(a)** perceived utilitarian value.**(b)** perceived emotional value.**(c)** travel intention on the spot.

Perceived utilitarian value is a manifestation of trusting and adopting the content scheme put forward by the tourism live streamer. In the specific context of online shopping, utilitarian value refers to the degree to which the function, price and quality provided by a product or service are consistent with the expected utility of consumers. When consumers’ expectations are fully met, they can experience the utilitarian value of products and services [38]. Some studies have found that utilitarian value has a positive impact on users’ intention to use and repeat purchases [59,60]. Exploring how to improve the authenticity of tourist travel intention through travel live streaming, the perceived value will let us see how travel live streaming can adapt to the individual needs of each tourist and provide them with differentiated value. When consumers find that travel live streaming provides them with valuable information, culture and services, they may have the demand for field travel.

**Hypothesis 6a.** Tourists’ perceived utilitarian value positively affects tourist travel intention on the spot.

Emotion is an individual’s subjective perception after contact with external people and things. According to the individual’s emotional attitude towards objects, perception can be divided into positive attitude and negative attitude. Through travel live streaming, the sense of existence and trust in perceived value can enhance consumers’ real intention to travel [48]. Positive emotional attitudes will have a positive impact on tourist travel intention, and these emotional attitudes can be divided into three dimensions [27]. If tourists have established a good interactive relationship with live streamer and have a strong interest in the destination, they are more likely to visit the place in person.

**Hypothesis 6b.** Tourists’ perceived emotional value positively affects tourist travel intention on the spot.

Kim & Lennon pointed out based on the extended SOR theory that consumers’ perceived risk has a negative impact on their emotions to a certain extent [61]. When tourists feel high risk, they will experience negative and negative emotions, and their intention to shop online will also decrease. Bitner [11] found based on SOR theory that when consumers perceive the high service quality of theme restaurants, they will have the will to participate again. Studies have shown that emotional participation plays an intermediary role between telepresence and impulse purchase [14], perceived utilitarian value also mediates the relationship between live streaming and tourist participation [15]. Based on the previous literature research, it can be predicted that perceived value plays the following intermediary role in this study.

**Hypothesis 7.** Perceived utilitarian value plays an intermediary role between **(a)** live streamer characteristics and travel intention.**(b)** live streaming content characteristics and travel intention.

**Hypothesis 8.** Perceived emotional value plays an intermediary role between **(a)** live streamer characteristics and travel intention.**(b)** live streaming content characteristics and travel intention.

**Fig 1:**
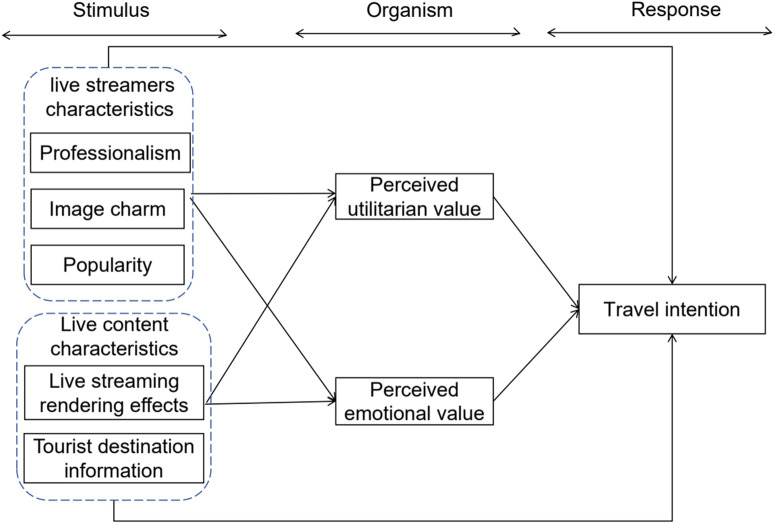
The research conceptual framework.

Therefore, the development model constructed in this study is as follows Fig 1 shown:

## 3. Research methods

### 3.1 Research in brief

This study aims to investigate how travel live streamers’ characteristics, live-streaming content attributes, and perceived value jointly influence viewers’ travel intention. A quantitative survey was developed comprising 28 items measured on a 7-point Likert scale. Eight constructs were included: streamer professionalism, image attractiveness, popularity, live-streaming visual appeal, destination-related information, perceived utilitarian value, perceived emotional value, and travel intention. All items were adapted from established domestic and international scales and refined with high-frequency keywords identified in prior qualitative work.

Data were collected online via WeChat, QQ, and TikTok between 8 October and 18 December 2023 using random sampling. Of 546 responses, 511 were valid (effective response rate = 93.6%), satisfying the 5-to-10 rule for sample size adequacy. The sample was gender-balanced; 46.2% held a high-school/technical-secondary education or below, 67.5% were corporate employees, and 42.3% watched travel live streams once or twice per month.

Reliability and exploratory factor analyses were conducted with SPSS 26.0. Cronbach’ s α for all constructs exceeded 0.70, and the overall KMO value was 0.924 (p < 0.001), with 77.77% cumulative variance explained, demonstrating satisfactory reliability and validity. Confirmatory factor analysis and structural equation modeling will be performed using AMOS 26.0 to test the hypothesized model and examine mediating effects among the variables.

### 3.2 Measures

In this study, questionnaire survey is used for quantitative research. Questionnaire survey is a data collection method often used in social surveys, which has direct and objective characteristics. The questions in the questionnaire are combined with the previously collected index dimensions and related maturity scales at home and abroad (See Table 4), using the Likert scale, from “1” to “7”, the degree of agreement gradually increased, and finally the questionnaire was formed. The questionnaire includes 8 dimensions. In terms of the characteristics of tourism live streamer, professionalism refers to the communicator professionalism measurement scale of Mengfei [62], while image charm is measured by high-frequency keywords such as “ideas”, “patterns”, and “reserves” mentioned above, combined with the measurement scale of Eisend & Langner [63]. Popularity refers to the measurement of reputation and prestige in Tong [51] scale, and combines keywords such as “Dean Zhang” and “fans”. In terms of the characteristics of live streaming content, live streaming rendering effect mainly refers to Zhang Chubing et al. [64] visual attraction scale, while tourist destination information refers to Dong Quansong [65] tourism resources condition measurement scale. In terms of intermediary variables, this paper refers to Sweeney & Soutar [66] measurement scale of perceived usefulness, the value of perceived emotion can be used for reference Zhou Yongsheng et al. [67] ognitive trust scale and Komiak & Benbasat [68] emotional scale. In terms of travel intention, this study refers to Dodds et al. [69] and Hu Fusheng [70] scholars’ research and measurement scale on intention.

**Table 4:** Variable design and sources.

Variable	Item	Description	Relevant sources
Professionalism	ZY1	Travel live streamer have good language skills in live streaming.	Mengfei [62]
	ZY2	Travel live streamer can create a good interactive atmosphere in live streaming.	
	ZY3	Travel live streamer can solve the questions and needs of tourists in a timely manner in live streaming.	
	ZY4	Travel live streamer give a comprehensive introduction to the prices of tourist destinations in live streaming.	
	ZY5	Travel live streamer give a comprehensive introduction to the route plan of the tourist destination in live streaming.	
	ZY6	Travel live streamer give a comprehensive explanation of the tourist attractions in live streaming.	
Image charm	XM1	Travel live streamer have a good image in live streaming (confident and generous).	Eisend & Langner [63]
	XM2	Travel live streamer have a talent show (singing and dancing) in live streaming.	
	XM3	Travel live streamer have a strong cultural literacy in live streaming (have a certain knowledge base).	
	XM4	The explanation of travel live streamer in live streaming reflects a certain ideological realm (there is a pattern, and the horizon is broad).	
Popularity	ZM1	Travel streamers are influential on social media platforms.	Tong [51]
	ZM2	Travel streamers are influential in the field.	
	ZM3	Travel streamers have a large number of fans, followers.	
Live streaming rendering effects	CX1	The content of the travel live streaming is clear and smooth.	Zhang Chubing et al. [64]
	CX2	The sound quality of the live tour is good, and there is no noise.	
	CX3	Travel live streaming will use multiple angles and different filming techniques to show the tourist destination.	
Tourist destination information	LX1	Travel live streaming showcase a variety of local attractions.	Dong Quansong [65]
	LX2	Travel live streaming showcase local snacks and delicacies.	
	LX3	Travel live streaming showcase various local tourism activities.	
Perceived utilitarian value	GJ1	Travel live streaming allow me to learn about various tourist destinations and attractions, avoiding the actual travel expenses and time costs.	Sweeney & Soutar [66]
	GJ2	Travel live streaming allow me to grasp more comprehensive tourism information, reduce the cost of information search, and improve the efficiency of tourism decision-making.	
	GJ3	Travel live streaming have given me a valuable experience in terms of self-awareness, cultural identity, entertainment and fun.	
Perceived emotional value	QJ1	Travel live streaming make me feel happy and relaxed.	Zhou Yongsheng et al. [67];Komiak & Benbasat [68]
	QJ2	Travel live streaming can spark my curiosity.	
	QJ3	The destination of the live tour made me feel reliable.	
Travel intention	LY1	After watching the live stream of the tour, it strengthened my idea of going to the field.	Dodds et al. [69];Hu Fusheng [70]
	LY2	After watching the live stream of the tour, it strengthened my desire to revisit the old place.	
	LY3	After watching the live stream of the tour, I will recommend others to travel to the destination.	

### 3.3 Data collection

In accordance with the regulations, this study is classified as a routine educational quality project. Therefore, it does not require approval from an ethics committee or institutional review board. This study does not involve animal or human clinical trials, nor is it unethical. In line with the ethical principles outlined in the Declaration of Helsinki, informed consent was obtained from all participants prior to their participation in the study. Participants’ anonymity and confidentiality are guaranteed, and participation was entirely voluntary.

This study used an online questionnaire to collect data on audiences of travel live streaming platforms in China. To reach a diverse audience, the questionnaire was distributed via multiple online platforms, including WeChat, QQ, and TikTok. Random sampling was used to identify participants, ensuring broad representation of viewers with varying levels of engagement with travel live streaming. Data was collected from October 8 to December 18, 2023.

### 3.4 Sample characteristics

The demographic information of the respondents is shown in Table 5. The proportion of men and women is close. In terms of the highest academic qualifications, senior high school/technical secondary school or below has the largest number, accounting for 46.2%, which is basically consistent with the current occupation ratio, and the proportion of enterprise employees is as high as 67.5%. In terms of the frequency of watching travel live streaming, 42.3% people watch it once or twice a month, which shows that it is a universal viewing frequency. These people may be interested in the tourism content.

**Table 5:** Demographic profile of respondents(n = 511).

Group		Frequency	Percentage
Gender	Male	271	53
	Female	240	47
Education	High school/technical secondary school and below	236	46.2
	Diploma	150	29.4
	Bachelor’s degree	101	19.8
	Master’s degree or above	24	4.7
Occupation	Student	43	8.4
	Corporate Employees	345	67.5
	Civil servants or personnel of public institutions	36	7%
	Self-employed or freelancers	80	15. 7
	Others	seven	1.4
Monthly income	Under 2000	43	8.4
	2000-5000	172	33.7
	5000-8000	183	35.8
	8000 or above	113	22.1
Frequency of watching travel live streaming	Rarely	92	18
	Once or twice a month	216	42.3
	Once or twice a week	116	22.7
	Every day	87	17

### 3.5 Data analysis

A total of 546 responses were received. After excluding incomplete and inconsistent responses, 511 were deemed valid, resulting in an effective response rate of 93.6%. This sample size met the requirement that the number of respondents should be 5–10 times the number of questionnaire items [71]. The reliability analysis and exploratory factor analysis (EFA) of 28 questions in the questionnaire were carried out by SPSS26.0, and the Cronbach’s Alpha coefficients of each variable and its dimension were all greater than 0.7 [72], which shows that the questionnaire has good reliability. KMO and bartlett spherical test are used to test the validity of the questionnaire. It is generally considered that KMO value is between 0.5 and 0.9, which is an acceptable range. The KMO test value of the overall survey data of this questionnaire is 0.924 (> 0.50), and the significance probability is 0.000 (p < 0.001). The load of each measurement item is higher than 0.5, indicating that each dimension is highly correlated with variables [73]. The total variance explanation rate of 8 factors is 77.769%, which is higher than the general standard of 60%. Due to the model’s complexity, AMOS 26.0 software was used to perform confirmatory factor analysis (CFA) and structural equation modeling (SEM) to test the structural characteristics of the conceptual model and analyze the significant mediating effects among the variables in the data.

## 4. Findings and discussion

### 4.1 Confirmatory factor analysis

**Fig 2:**
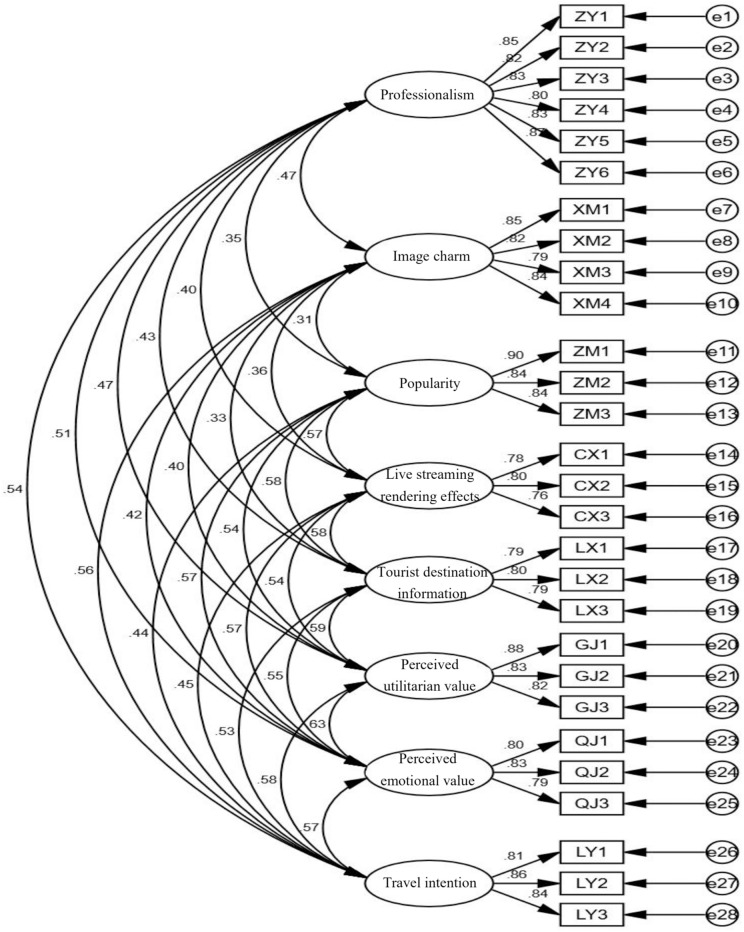
Model diagram of confirmatory factor analysis.

The results of CFA are shown and summarized in Table 6 and Fig 2. χ2/df = 1.891(χ2/df < 3); RMSEA = 0.042 (RMSEA< 0.08), and the result of this study accords with Kline (2010) and Wu (2009). CFI = 0.970 (threshold level > 0.90), IFI = 0.970 (threshold level > 0.90) and TLI = 0.964 (threshold level > 0.90), which are all satisfactory [74]. GFI = 0.922, AGFI = 0.902 and NFI = 0.938, all above the critical value of 0.9 [72,75]. To sum up, the overall goodness of fit index of the measurement model is accepted.

**Table 6:** Confirmatory factor analysis results (N = 511).

Variable	Measured item	Standardized loading	S.E.	C.R.	P	CR	AVE
Professionalism	ZY1	0.852				0.915	0.683
	ZY2	0.817	0.041	22.995	***		
	ZY3	0.826	0.041	23.425	***		
	ZY4	0.803	0.041	22.383	***		
	ZY5	0.834	0.04	23.805	***		
	ZY6	0.87	0.039	25.589	***		
Image charm	XM1	0.855				0.896	0.683
	XM2	0.825	0.042	22.293	***		
	XM3	0.787	0.042	20.831	***		
	XM4	0.837	0.044	22.785	***		
Popularity	ZM1	0.901				0.895	0.739
	ZM2	0.841	0.038	24.507	***		
	ZM3	0.836	0.036	24.297	***		
Live streaming rendering effects	CX1	0.776				0.823	0.609
	CX2	0.8	0.055	16.961	***		
	CX3	0.764	0.06	16.36	***		
Tourist destination information	LX1	0.787				0.836	0.629
	LX2	0.805	0.055	17.844	***		
	LX3	0.788	0.055	17.521	***		
Perceived utilitarian value	GJ1	0.881				0.883	0.715
	GJ2	0.835	0.04	23.108	***		
	GJ3	0.819	0.039	22.508	***		
Perceived emotional value	QJ1	0.803				0.850	0.653
	QJ2	0.834	0.05	19.481	***		
	QJ3	0.787	0.052	18.418	***		
Travel intention	LY1	0.808				0.874	0.699
	LY2	0.861	0.051	21.181	***		
	LY3	0.838	0.05	20.631	***		

Composite reliability value can be used to test the reliability of internal consistency, and the composite reliability value should be greater than 0.7 [76]. As shown in Table 6, the composite reliability values of all potential variables are between 0.823 and 0.915, which are higher than the critical value of 0.7. The factor loads of all 28 projects are above 0.5, ranging from 0.764 to 0.901 [77]. In addition, the AVE values of all potential variables are between 0.609 and 0.739, which all exceed the threshold level of 0.5 [78].

The discriminant validity of all constructs is tested by the square root of AVE (see Table 7). The correlation coefficient in discriminant validity should be controlled within the critical value of 0.85 [79]. As can be seen from Table 7, it can be seen that the variance of AVE value of each variable is greater than the correlation coefficient between each variable, which means that it has good discrimination validity [78]. Generally speaking, the above discussion shows that the reliability of internal consistency, convergence validity and discrimination validity are acceptable.

**Table 7:** Results of discriminant validity (N = 511).

Construct	1	2	3	4	5	6	7	8
1 Professionalism	0.827							
2 Image charm	0.422	0.826						
3 Popularity	0.321	0.278	0.860					
4 Live streaming rendering effects	0.35	0.313	0.493	0.780				
5 Tourist destination information	0.382	0.294	0.498	0.483	0.793			
6 Perceived utilitarian value	0.43	0.36	0.489	0.466	0.508	0.845		
7 Perceived emotional value	0.453	0.362	0.495	0.478	0.464	0.548	0.808	
8 Travel intention	0.495	0.5	0.393	0.391	0.455	0.511	0.493	0.836

Note: The bold diagonal values are the square roots of AVE, and the non-bold off-diagonal values are latent variable correlations.

### 4.2 Structural equation model

Using AMOS 26.0 to establish a structural equation model to test the relationship between independent variables and dependent variables. The results show that all the goodness-of-fit indexes in the structural equation model established in this study have reached and exceeded the general standard values, and the overall goodness-of-fit indexes are acceptable (χ2/df = 1.957, RMSEA = 0.043, CFI = 0.967, IFI = 0.968, TLI = 0.962 [74,79,80], GFI = 0.920, AGFI = 0.899, NFI = 0.936 [72,75].

#### 4.2.1 Direct road inspection.

The correlation results of path regression coefficient and significance test obtained by SEM using AMOS26.0 are shown in Table 8. According to Hair, Hult, Ringle, and Sarstedt [81], the t-value is greater than 1.96 at 5% significance level, because when t-value is greater than 1.96, p-value is just less than 0.05 significance level, and if t-value is less than 1.96, p-value of significance cannot reach significance level.

**Table 8:** Results of direct effects (N = 511).

Hypothesis	Path	Standard coefficient	S.E.	C.R.	P-value	Result
H1a	Professionalism →Perceived utilitarian value	0.163	0.045	3.462	***	Support
H1b	Professionalism →Perceived emotional value	0.212	0.04	4.399	***	Support
H2a	Image charm →Perceived utilitarian value	0.114	0.051	2.491	0.013	Support
H2b	Image charm →Perceived emotional value	0.112	0.044	2.411	0.016	Support
H3a	Popularity →Perceived utilitarian value	0.195	0.06	3.576	***	Support
H3b	Popularity →Perceived emotional value	0.24	0.053	4.304	***	Support
H4a	Live streaming rendering effects →Perceived utilitarian value	0.183	0.069	3.139	0.002	Support
H4b	Live streaming rendering effects →Perceived emotional value	0.226	0.061	3.764	***	Support
H5a	Tourist destination information →Perceived utilitarian value	0.27	0.076	4.497	***	Support
H5b	Tourist destination information →Perceived utilitarian value	0.164	0.065	2.726	0.006	Support
H1c	Professionalism →Travel intention	0.174	0.039	3.587	***	Support
H2c	Image charm →Travel intention	0.286	0.043	6.104	***	Support
H3c	Popularity →Travel intention	0.022	0.051	0.391	0.696	Not supported
H4c	Live streaming rendering effects →Travel intention	−0.012	0.059	−0.208	0.835	Not supported
H5c	Tourist destination information →Travel intention	0.157	0.064	2.557	0.011	Support
H6a	Perceived utilitarian value →Travel intention	0.191	0.047	3.341	***	Support
H6b	Perceived emotional value →Travel intention	0.154	0.059	2.538	0.011	Support

*p < 0.05,**p < 0.01, ***p < 0.001

In terms of direct path, the research results support 15 hypotheses: H1a (t = 3.462, p < 0.001), H1b (t = 4.399, p < 0.001), H2a (t = 2.491, p < 0.05), H2b (t = 2.411). p < 0.001), H4a (t = 3.139, p < 0.05), H4b (t = 3.764, p < 0.001), H5a (t = 4.497, p < 0.001), H5b (t = 2.726, P < 0.001), H5c (t = 2.557, p < 0.05), H6a (t = 3.341, p < 0.001), H6b (t = 2.538, p < 0.05). These data show that professionalism, image charm, popularity, live streaming rendering effect and tourist destination information all have positive effects on perceived utilitarian value and perceived emotional value, while professionalism, image charm, tourist destination information, perceived utilitarian value and perceived emotional value all have positive effects on field travel intention.

#### 4.2.2 Indirect road inspection.

In this study, Bootstrap method is used to explore the intermediary role of the two constructions of perceived utilitarian value and perceived emotional value in the research model. Select 95% confidence interval, and then calculate and test the mediation effect through 5000 rotation iterations built into the software. The research results are shown in Table 9.

**Table 9:** Results of indirect effects (N = 511).

Indirect paths	Estimate	Lower	Upper	P-value
Professionalism →Perceived utilitarian value →Travel intention	0.031	0.007	0.072	0.004
Image charm →Perceived utilitarian value →Travel intention	0.022	0.003	0.058	0.018
Popularity →Perceived utilitarian value →Travel intention	0.037	0.01	0.086	0.003
Live streaming rendering effects →Perceived utilitarian value →Travel intention	0.035	0.01	0.082	0.003
Tourist destination information →Perceived utilitarian value →Travel intention	0.051	0.016	0.107	0.003
Professionalism →Perceived emotional value →Travel intention	0.033	0.007	0.074	0.01
Image charm →Perceived emotional value →Travel intention	0.017	0.002	0.051	0.02
Popularity →Perceived emotional value →Travel intention	0.037	0.009	0.086	0.009
Live streaming rendering effects →Perceived emotional value →Travel intention	0.035	0.007	0.084	0.009
Tourist destination information →Perceived emotional value →Travel intention	0.025	0.003	0.072	0.015

According to Table 9, each path is positive in the upper and lower 95% confidence interval, excluding 0, and the significant p-value is < 0.05, which shows that the mediation effect is significant, so the hypothesis put forward in this study is verified [82], perceived utilitarian value and perceived emotional value play an intermediary role in professionalism, image charm, popularity, live streaming rendering effect, tourism destination information and field travel intention.

## 5. Discussion and meaning

As a new trend in the tourism industry, Gefen and Straub [83] believed that live streaming can give the audience a sense of existence, which can increase and consolidate the interaction between members and enhance trust [8]. Although the research on live streaming has developed rapidly in recent ten years, many studies only focus on the role of live streaming marketing strategy. Previous studies used live streaming to explore the relationship between e-commerce and consumer behavior choices (such as: Clement Addo et al. [84]), there are also studies that use travel live streaming to attract potential tourists (such as: Zhang et al. [4], Li et al. [6] and Zheng et al. [48]), but people know little about how the characteristics of travel live streaming affect tourist travel intention. Therefore, it is necessary to study the influence factors of the characteristics of travel live streaming on tourist travel intention on the spot. Tourism can also satisfy tourists’ sense of pleasure and value, so it is necessary to study tourist travel intention on the spot from the perspective of perceived value. The variables in this study are mainly derived from text mining, and the more important variables are extracted from the user’s perspective, so the two important dimensions of function and emotional value are discussed in detail from data processing. It is undeniable that while other common dimensions such as social and cognitive may also help identify external influences and explain internal psychological mechanisms, their manifestations were not sufficiently prominent in our data processing; thus, they were not emphasized in the final analysis. Therefore, this study constructs a theoretical model based on SOR theory, which consists of live streamer characteristics (professionalism, image charm and popularity), live streaming content characteristics (live streaming rendering effect, tourist destination information), perceived emotional value and perceived utilitarian value, and intention to travel on the spot.

This study adopts a combination of qualitative and quantitative methods, and at the same time following the recommendations of Churchill [85] and DeVellis [86], to determine a new research scale containing 28 items to measure the complex interaction relationships between variables. In addition, this study determines three dimensions of live streamer characteristics through text mining: professionalism, image charm and popularity, which is consistent with previous studies [4,5]. At the same time, two dimensions of live streaming content characteristics are determined: live streaming rendering effect and tourist destination information, which are also consistent with the variables studied by predecessors [5,87].

The results of the structural equation model show that live streamer professionalism and image charm, as well as tourist destination information and perceived value in live streaming content characteristics, directly affect tourist travel intention. Additionally, the characteristics of live streamers and live content in travel live streaming directly affect users’ perceived emotional and utilitarian values. Furthermore, perceived value plays an intermediary role between live streamer and live content characteristics and travel intention. Compared to previous related studies, this study yielded new findings. First,the novelty and uniqueness of this study primarily lie in its adoption of text mining methods to explore the factors of interest in tourism live streams from the tourists’ perspective which extract travel live streaming characteristics from obtained texts and combines text mining with a questionnaire survey to expand research methods. Second, previous studies have shown that live streamer and live streaming content characteristics impact consumers’ intention to act, but these studies have mainly focused on e-commerce live streaming. This study expands the positive influence of these two dimensions on tourists’ intentions in the tourism field. Third, the results of this study contribute to related research on perceived emotional and utilitarian values and travel intention. Finally, this study discusses the relationship between perceived emotional and utilitarian values and travel live streaming’s impact on travel intention. The results show that perceived emotional and utilitarian values mediate the relationship between travel live streaming and travel intention. Additionally, the results show a double intermediary effect: the characteristics of the live streamer and content affect travel intention through emotional and utilitarian value perception. Therefore, this study has theoretical and practical significance.

### 5.1 Theoretical contribution

Based on the empirical results, this study provides several theoretical contributions.

First of all, this study uses the method of text mining to dig out five dimensions of the characteristics of travel live streaming from the network environment, and through the combination of qualitative and quantitative methods, verifies and enriches the methods of measuring tourists’ behavior by the characteristics of travel live streaming. Although live streamer plays an important role in live streaming [4], there are also scholars who study the factors that affect consumers by live content [21], live streaming platform also has more purchase intentions [2] and intention to participate [3], but the previous research in the field of tourism did not explore the dimensions of live streamer and live streaming content through text mining, and verify and enrich the related content of live streaming. In addition, this paper determines a 28-item scale to measure the characteristics of live streamer and live streaming content in travel live streaming, which synthesizes previous findings. Finally, this study verifies that there are many dimensions in travel live streaming: live streamer characteristics (professionalism, image charm and popularity) and live streaming content characteristics (live streaming rendering effect and tourist destination information). Therefore, the 28 scales used in this study and the five dimensions in travel live streaming can be used as reference tools and contents for future research to evaluate the influencing factors of tourists’ intentions.

The second theoretical contribution of this study is to determine the relationship between live streamer characteristics, live streaming content characteristics, perceived utilitarian value and perceived emotional value, and tourist travel intention on the spot in the tourism field. First, the professionalism and image charm of live streamer characteristics have a positive impact on travel intention on the spot, which is consistent with the previous research literature [35,51]. Popularity has no significant effect on travel intention, which is related toLee & Thorson [55] and Zhang et al. [56] the findings are inconsistent, which shows that although tourism live streamer are well-known, they may be regarded as more representatives of brand image, and the traffic brought by tourists’ interest in live streaming of well-known live streamer may be more “onlookers” than potential tourists with clear tourism purposes. In addition, due to the randomness and instantaneous nature of tourists’ behavior, tourists jump between multiple live streaming when watching live streaming, and can’t continue to pay attention to a live streamer, which leads to the weakening of the influence and persuasiveness of live streamer. In addition, this paper also finds that the tourism destination information in the characteristics of live streaming content has a positive impact on travel intention on the spot [9] (consistent with Zheng et al.[48]), but the presentation effect has no significant impact on travel intention (inconsistent with Ye et al. [23] and Sang et al.[58]), which may indicate that although the presentation effects of the technical aspects such as the clarity, fluency and sound quality of live streaming content have a direct impact on the instant viewing experience of tourists, this does not guarantee that tourists will turn this positive experience into actual travel actions. Secondly, this study found that both live streamer characteristics and live streaming content characteristics have a positive impact on the perceived utilitarian value and emotional value. The results of this study are helpful to supplement the existing related research, because the previous research did not explore the relationship between tourism live streamer and content characteristics on perceived utilitarian value and perceived emotional value. People watch live streaming to learn about the relevant information of tourist destinations [9], you can feel relaxed, happy and valuable in live streaming, and help tourists make better choices. In this sense, tourists who feel high value can meet their needs for information by watching live streaming, and they will also have corresponding positive behaviors in their choices. Thirdly, there is a positive correlation between perceived emotional value and perceived utilitarian value on travel intention. When the user is faced with a choice, the higher the perceived value, the more interested the user is in buying a product [11], because it creates a sense of satisfaction for users. Scholars have studied the relationship between perceived value and consumers’ intention to choose behavior [59,60], and this study clearly divides perceived value into two dimensions: perceived emotional value and perceived utilitarian value. However, the dimension of perceived value is still developing, and this dimension may not be fully used. The relationship between perceived emotional value, perceived utilitarian value and travel intention has been verified for the first time in the field of tourism. Tourists with high perceived value can usually make more accurate choices, and they will share the information obtained in live streaming room in order to convey the information to other tourists.

Thirdly, this study reveals that two variables, perceived utilitarian value and perceived emotional value, play an indispensable and important role in the relationship between the characteristics of travel live streaming and travel intention. The findings of this study enrich previous studies, because in the past, scholars studied perceived value from different aspects, such as utility, emotion, integrity, cost performance, sociality, novelty, economy, society and so on [12,38–40], without taking perceived utilitarian value and perceived emotional value as double mediating variables, this paper discusses their mediating role between the characteristics of travel live streaming and travel intention. In addition, by comparing the influence coefficients, this study finds that perceived utilitarian value plays the most significant mediating role. Therefore, this study discusses the important position of perceived utilitarian value and perceived emotional value in the process of tourists’ decision-making, and expands the related research of tourists’ travel intention to a certain extent in the future, which has good theoretical significance.

### 5.2 Practical value

From a practical point of view, this study provides business suggestions for tourism stakeholders, such as live streamers and live streaming platforms, in the process of selecting a live streamer. First, the professionalism and charm of travel live streamers have an important influence on tourists’ travel intentions. As a brand-new marketing method for internet platforms, travel live streaming combines online and offline functions to disseminate information and promote products. The demand for professional talent in this new field is growing, and China is facing talent challenges in travel live streaming. At the same time, the professionalism and image charm of tourism live streamers can directly affect tourists’ travel intentions. In light of this finding, managers and operators of tourist attractions must pay attention to systematically training live streaming talent. For example, they can strengthen the professional knowledge training of tourism live streamers so that they can accurately introduce the historical background, cultural characteristics, and key points of tourist attractions. This will enhance the authority and ornamental value of the live streaming content. Second, live streamers can highlight the unique features of tourism products and destinations and design travel routes to attract tourists. They can also provide interesting information to tourists by interacting with them, allowing tourists to access a travel guide at any time. Finally, cultivate the personal image and charm of the live streamer. Encouraging the live streamer to showcase their talents, such as singing, playing instruments, and painting, can increase interest in live streaming and build a unique personal brand. This approach can also leverage word-of-mouth communication to garner more attention from tourists. At the same time, expanding the cultural knowledge of live streamers allows them to flexibly use various cultural elements in live streaming, thereby enriching the content and improving its educational and entertainment value.

The second factor that directly affects tourists’ travel intentions is the relevant tourist destination information in live-streaming content. This information provides effective analysis and guidance for the sustainable development of tourist destinations in the future. This information includes various local attractions, specialty snacks, and tourism activities displayed in travel live streams. Research shows that tourists are more willing to watch content about scenic spots and various activities and foods, which stimulates their curiosity and desire to explore, producing a stronger travel intention. Therefore, during live streaming, the live streamer should focus on relevant content. Before live streaming, the live streamer should release information about the scenic spots in the form of pictures, text, and audio so that more people can learn about the spots and their characteristic culture. At the same time, scenic spots can hold tourism salons, organize tourism lectures, hold tourism experience camps, hold various prize-winning quizzes, and set up food festivals featuring local cuisine, etc., to increase travel intentions.

Finally, the perceived emotional and utilitarian values of intermediaries cannot be ignored. Therefore, it is important for tourism managers and operators to strengthen interaction and contact between tourists during travel live streaming. In addition to the above two factors, we must also consider the popularity of the live streamer and the rendering effect of the live stream, as these can increase the perceived value of tourists and thus affect their choice of destinations. For example, when selecting a live streamer, invite famous or popular stars to visit the live streaming room. Additionally, enhance the live streaming viewing experience by ensuring a smooth picture, stable signal, synchronized audio and video, clear image quality, and real-time interactivity with minimal delay. Upgrade live streaming equipment and leverage advanced technologies such as 5G network technology, 4K Ultra HD, VR, and AI to enhance the viewing experience and ensure tourists are deeply immersed in the charm of live streaming.

## 6. Limitations and future research

Although this study has certain theoretical and practical significance, there are still some limitations that must be addressed to improve the efficiency of future research. First, this study’s sample only tested live streaming, perceived value, and travel intention regarding the world tour. It focused on comments from live streams of the world tour selected by the East. In the contextual discussion of this research, fundamental differences in content logic, user demographics, and interaction modes across various live streaming platforms directly determine the “influence pathways” (e.g., information acquisition, decision triggering, behavioral conversion) and “intensity of impact” (e.g., seed-planting effect, trust level) of tourism live streams on tourists. Cultural differences between countries can also have very different effects. This study selects social platforms widely used by Chinese users, which are more in line with Chinese user habits and culture. In the future, the attitudes and reactions of different countries can be selected to explore the effects of cultural differences. Although the respondents said they watched live streams on multiple platforms, the results may still be biased. Therefore, future research should collect text from various travel live-streaming videos to expand the text data related to travel live-streaming. Second, this study primarily used questionnaires to collect data, and the responses are more subjective. While filling out the questionnaires, respondents may be influenced by their emotions, environment, and travel experience, resulting in subjective answers that affect the analysis results. In-depth interviews and questionnaires can then be combined to further ensure the accuracy of the results. Third, in practice, many tourists may intend to travel but do not actually travel, which differs from those who intend to travel and take action. Therefore, future research should target tourists who have travel intentions and have already taken action. Fourth, this study’s data is from Chinese people, so the results may not represent the tourism situation in other countries or regions. Future research can collect data from tourists in other countries or regions to verify these findings. Finally, this study only focuses on tourists who watch travel live streaming. Future research should also explore other stakeholders, such as the government, tourism departments, tourism enterprises, and advertisers. More in-depth related research is needed.

## Supporting information

S1 Data Minimum data set. (RAR)
